# Decoding disparities: evaluating automatic speech recognition system performance in transcribing Black and White patient verbal communication with nurses in home healthcare

**DOI:** 10.1093/jamiaopen/ooae130

**Published:** 2024-12-10

**Authors:** Maryam Zolnoori, Sasha Vergez, Zidu Xu, Elyas Esmaeili, Ali Zolnour, Krystal Anne Briggs, Jihye Kim Scroggins, Seyed Farid Hosseini Ebrahimabad, James M Noble, Maxim Topaz, Suzanne Bakken, Kathryn H Bowles, Ian Spens, Nicole Onorato, Sridevi Sridharan, Margaret V McDonald

**Affiliations:** Columbia University Irving Medical Center, New York, NY 10032, United States; School of Nursing, Columbia University, New York, NY 10032, United States; Center for Home Care Policy & Research, VNS Health, New York, NY 10017, United States; Center for Home Care Policy & Research, VNS Health, New York, NY 10017, United States; School of Nursing, Columbia University, New York, NY 10032, United States; Columbia University Irving Medical Center, New York, NY 10032, United States; Columbia University Irving Medical Center, New York, NY 10032, United States; Department of Computer Science, Columbia University, New York, NY 10027, United States; School of Nursing, Columbia University, New York, NY 10032, United States; Department of Automatic Control and Computer Science, Politehnica University of Bucharest, Bucharest RO-060042, Romania; Columbia University Irving Medical Center, New York, NY 10032, United States; Department of Neurology, Taub Institute for Research on Alzheimer’s Disease and the Aging Brain, GH Sergievsky Center, Columbia University, New York, NY 10032, United States; Columbia University Irving Medical Center, New York, NY 10032, United States; School of Nursing, Columbia University, New York, NY 10032, United States; Center for Home Care Policy & Research, VNS Health, New York, NY 10017, United States; Data Science Institute, Columbia University, New York, NY 10027, United States; School of Nursing, Columbia University, New York, NY 10032, United States; Data Science Institute, Columbia University, New York, NY 10027, United States; Department of Biomedical Informatics, Columbia University, New York, NY 10032, United States; Center for Home Care Policy & Research, VNS Health, New York, NY 10017, United States; University of Pennsylvania School of Nursing, Philadelphia, PA 19104, United States; Center for Home Care Policy & Research, VNS Health, New York, NY 10017, United States; Center for Home Care Policy & Research, VNS Health, New York, NY 10017, United States; Center for Home Care Policy & Research, VNS Health, New York, NY 10017, United States; Center for Home Care Policy & Research, VNS Health, New York, NY 10017, United States

**Keywords:** automatic speech recognition (ASR), home healthcare, health disparities, word error rate (WER), speech to text, linguistic inquiry and word count (LIWC)

## Abstract

**Objectives:**

As artificial intelligence evolves, integrating speech processing into home healthcare (HHC) workflows is increasingly feasible. Audio-recorded communications enhance risk identification models, with automatic speech recognition (ASR) systems as a key component. This study evaluates the transcription accuracy and equity of 4 ASR systems—Amazon Web Services (AWS) General, AWS Medical, Whisper, and Wave2Vec—in transcribing patient-nurse communication in US HHC, focusing on their ability in accurate transcription of speech from Black and White English-speaking patients.

**Materials and Methods:**

We analyzed audio recordings of patient-nurse encounters from 35 patients (16 Black and 19 White) in a New York City-based HHC service. Overall, 860 utterances were available for study, including 475 drawn from Black patients and 385 from White patients. Automatic speech recognition performance was measured using word error rate (WER), benchmarked against a manual gold standard. Disparities were assessed by comparing ASR performance across racial groups using the linguistic inquiry and word count (LIWC) tool, focusing on 10 linguistic dimensions, as well as specific speech elements including repetition, filler words, and proper nouns (medical and nonmedical terms).

**Results:**

The average age of participants was 67.8 years (SD = 14.4). Communication lasted an average of 15 minutes (range: 11-21 minutes) with a median of 1186 words per patient. Of 860 total utterances, 475 were from Black patients and 385 from White patients. Amazon Web Services General had the highest accuracy, with a median WER of 39%. However, all systems showed reduced accuracy for Black patients, with significant discrepancies in LIWC dimensions such as “Affect,” “Social,” and “Drives.” Amazon Web Services Medical performed best for medical terms, though all systems have difficulties with filler words, repetition, and nonmedical terms, with AWS General showing the lowest error rates at 65%, 64%, and 53%, respectively.

**Discussion:**

While AWS systems demonstrated superior accuracy, significant disparities by race highlight the need for more diverse training datasets and improved dialect sensitivity. Addressing these disparities is critical for ensuring equitable ASR performance in HHC settings and enhancing risk prediction models through audio-recorded communication.

## Introduction

Verbal communication between patients and nurses in home healthcare (HHC) settings is essential for uncovering significant health information, identifying signs of serious conditions, and revealing risk factors for adverse outcomes.^1^^,^^2^ Despite its importance, traditional methods of data collection often fail to capture this communication, leading to significant gaps in the availability of such data in electronic health records (EHRs). Research shows that up to 50% of clinical risk factors discussed during patient-nurse encounters remain undocumented, underscoring the need for improved data collection methods.^2^ To address this gap, we have developed a practical pipeline for audio recording of patient-nurse verbal communication in HHC. Integrating these audio recordings with EHR data significantly enhances the performance of risk identification models compared to those built solely on EHR data.^3^

Automatic Speech Recognition (ASR) technology, which transcribes spoken communication into text, has been explored to reduce clinical documentation burdens and improve information accuracy.^3^ Existing studies mostly focused on measuring accuracy of ASR in mental health settings, such as psychiatric interviews and psychotherapy.^3–6^ These studies typically involve patients under 45, and the corpora were transcribed from conversations controlled by the physicians to follow the specific screening or therapy process.^4^^,^^5^ However, ASR transcription accuracy varies significantly, with word error rates ranging from 18% to 63%, depending on the system and setting.^3^^,^^7^

In HHC, however, most patients are over 65 and often have physical or cognitive impairments.^8^^,^^9^ These vulnerabilities can affect their speech patterns and even ASR accuracy. In addition, these patients receive intermittent care from HHC clinicians, giving them open-ended opportunities to describe their recent progress and symptoms, which helps to facilitate the clinician’s assessment.^10^ Collectively, the uncertainty of ASR performances, combined with the uniqueness of HHC, underscore the need for a thorough evaluation of ASR, as its effectiveness in this setting remains uncertain.

While accurate transcription of nurse-patient interactions using ASR systems could improve the healthcare procedure, these systems are not uniformly effective.^11^ Research shows that ASR performs worse for racial and ethnic minorities, particularly speakers of African American vernacular English (AAVE).^11^^,^^12^ This underperformance is primarily due to the underrepresentation of AAVE in training datasets, such as the Texas Instruments/Massachusetts Institute of Technology corpus, which includes only 4% Black speakers.^11^ This presents a significant real-world challenge, as AAVE is spoken by nearly 80% of the Black population in the United States, amounting to approximately 35-40 million people.^13^

Previous studies have shown that AAVE differs from Standard American English in phonology, morphology, and syntax^12^^,^^14^^,^^15^: Phonologically, AAVE often features nonrhoticity, consonant cluster simplification, and th-stopping, where “th” is replaced with “d” or “t.” Morphologically, AAVE lacks some tense markers like the “-ed” for past tense and uses constructions like the habitual “be” for repeated actions. Syntactically, AAVE includes multiple negation (eg, “I don’t know nothing”) and zero copula, where “is” or “are” is omitted.

In HHC in New York city, where approximately 35% of patients are Black, many of whom speak AAVE, generating equitable ASR performance is critical.^16^ The disparities in ASR accuracy can lead to incomplete patient assessments and exacerbate the existing HHC inequities.^17^ Therefore, it is essential to reevaluate ASR systems across racial and ethnic subgroups in HHC to identify and mitigate bias. This will lay the foundation for future research using ASR systems to analyze HHC patient languages, paving the way for equitable care across diverse populations.

Therefore, this study aims to further analyze the performance of ASR system’s verbal communication of Black and White patient-nurse encounters in HHC as the foundation for enhancing integration of these data into EHR and influencing patient care. The first aim is to evaluate the accuracy of 4 commercial and open-source ASR systems in transcribing audio-recorded patient-nurse verbal communication in HHC settings. The second aim is to investigate potential differences in the accuracy of these systems when transcribing verbal communications between older adult patients and nurses, with a particular focus on differences between Black and White patients.

## Methods

This study was conducted at VNS Health, one of the largest not-for-profit HHC organizations in the United States, with Institutional Review Board (IRB) approval (IRB # E20-003). We recruited registered nurses specializing in older adult home care to record their communication with patients during home visits. Nurses introduced the study to interested patients, and a research assistant then obtained informed consent. Eligible patients had to be English-proficient, able to communicate independently with nurses, and cognitively capable of understanding and signing the consent without caregiver assistance.

### Procedure of audio recording

Through a set of pilot studies,^3–5^ we developed a practical method for audio recording of patient-nurse communication in HHC. The Saramonic Blink500 device was chosen for its usability and superior audio quality. This device includes 2 wireless microphones attached to participants’ clothing, transmitting audio to a recording device like an iPod, storing recordings in separate channels (see Appendix S1). Audio data were securely stored on Health Insurance Portability and Accountability Act of 1996 (HIPAA)-compliant AWS cloud servers. Using this method, we recorded the verbal communication of 47 patients: 16 Black, 19 White, and 12 of other races.

### Development of a gold standard transcription

To establish a gold standard (GS) for manual transcription in HHC, we employed purposeful sampling to select a representative subset of audio-recorded patient-nurse communication from a larger dataset. We chose the 10 longest recordings—5 with Black patients and 5 with White patients—featuring substantial patient engagement (where patients contributed significantly to the conversation by asking questions, providing detailed responses, or initiating topics), focused health discussions, and minimal background noise (limited ambient sounds like television, family conversations, or environmental noises that could interfere with audio clarity). Two HHC experts rigorously reviewed this selection to ensure it accurately represented typical patient-nurse interactions.

Creating a GS manual transcription of patient-nurse communication required proficiency in the dialects of both Black and White older adult patients, making external transcription services unsuitable for our study. These services often misinterpret terms, omit filler words like “um” and “uh,” and repetition of words or phrases, which are key indicators of speech fluency linked to cognitive impairment and mental disorders.^6^ Our analysis showed these services have a word error rate (WER) of 14% (see the section “Calculating ASR errors using WER” for WER definition), which is too high for reliable GS transcription.

To create a high-quality GS, we developed comprehensive guidelines for manual transcription to ensure precise and consistent documentation. Two research assistants with healthcare backgrounds and experience in HHC transcribed every utterance in patient-nurse communication verbatim, including all filler words and repetitions. The guidelines required standardized conversions for numerical and temporal expressions, such as “one hundred and fifty” to “150” and “a quarter past four” to “4:15.” To ensure quality and integrity, a third reviewer (S.V.), with similar qualifications, randomly audited 30% of the transcriptions and assisted with challenging segments. Weekly meetings were held to resolve discrepancies, resulting in an interrater agreement score of 0.83, indicating high transcription quality and reliability.

In total, 860 utterances were transcribed, with 475 utterances attributed to 5 Black patients and 385 to 5 White patients from the larger sample of the audio-recorded data. To maintain a focused and unbiased analysis of ASR accuracy within patient verbal communication, utterances by nurses were excluded.

### Automatic transcription of patients verbal communication

Automatic speech recognition systems are designed to convert spoken language into written text. Traditionally, many ASR systems consist of 2 primary components: an acoustic model and a language model.^7^ The acoustic model processes audio signals to generate probabilities over sequences of acoustic units—such as phonemes or other subword units—which are then used to form initial word hypotheses. The language model evaluates these hypotheses by assessing the likelihood and contextual appropriateness of word sequences, helping to select the most probable transcription. However, not all ASR systems decouple the process into distinct stages of audio-to-phoneme-to-text. Modern ASR systems increasingly utilize end-to-end neural network architectures that map audio signals directly to text outputs. These models integrate acoustic and linguistic processing into a single framework, optimizing transcription accuracy through joint training. OpenAI’s Whisper and Meta’s Wav2Vec are examples of such an end-to-end ASR system^8^ that does not explicitly decode audio into phonemes before generating text.

We employed 4 ASR systems from leading AI companies to automatically transcribe the sample of patient-nurse verbal communication in this study: 2 commercial systems, Amazon Web Services (AWS) General Transcribe^9^ (General) and AWS Medical Transcribe^10^ (AWS Medical), and 2 open-source systems, Whisper (Whisper-large-v3),^11^ and Wav2Vec 2.0.^12^ Whisper and Wav2Vec 2.0 can be installed on local servers, while AWS systems are accessed via an application programming interface as a cloud-based service. Amazon Web Services are HIPAA-compliant, and Amazon has partnerships with healthcare organizations across the United States.

Amazon ASRs use a speech recognition pipeline that combines hand-crafted features like mel-frequency cepstral coefficients^13^ with transformer-based deep learning models for both acoustic and linguistic processing. The acoustic model converts audio features into phonetic units, while the language model evaluates these units contextually to enhance word recognition accuracy. Additionally, a speaker diarization model distinguishes between speakers, improving transcription quality. Whisper employs a fully transformer-based architecture for end-to-end speech recognition, processing raw audio to text within transformer networks. In contrast, Wav2Vec uses self-supervised learning to extract features from raw audio, predicting masked segments from other segments and generating its own labels, allowing it to learn detailed speech representations from large amounts of unlabeled audio.

Amazon and Whisper ASRs were trained on extensive labeled audio datasets, while Wav2Vec was pretrained on large amounts of unlabeled audio data and fine-tuned with a smaller labeled dataset, such as LibriSpeech. Amazon Web Services Medical Transcribe is uniquely trained on extensive medical terminologies, enhancing its accuracy in specialized medical transcription. The differences in the architecture of the speech recognition pipeline and the type and size of the training datasets impact the performance of these ASR systems in transcribing HHC communication between Black and White patients and nurses. More information on the ASR systems is available in Appendix S1.

### Aligning patients’ utterances in GS with ASR transcription

To align the utterances in the GS with those in ASR systems, we implemented the following procedure:

1. Exclusion of Nurses’ Utterances: In the GS transcription, nurses’ utterances were manually excluded. For Amazon ASR transcriptions, these utterances were identified using speaker diarization, followed by manual annotation to designate speakers as patient or nurse. For Whisper, we used an open-access tool that employs voice activity detection^14^ and Whisper’s speaker embedding for diarization. For Wav2Vec, we applied diarization codes from its Hugging Face library^12^ and manually assigned speaker identities as patient or nurse.
2. Normalization of ASR transcriptions: We applied the tailored dictionary used for development of GS transcription to standardize numerical and temporal expressions in all ASR transcriptions, ensuring accuracy and consistency.
3. Aligning utterances in GS and ASR transcriptions: Aligning utterances between the GS and ASR transcriptions was challenging due to ASR systems sometimes missing short utterances, like “okay,” or those affected by unclear speech, and generating extra utterances due to background noise. We used a “word edit distance” algorithm^15^ to align the transcriptions at the word level by calculating the minimum word insertions, deletions, and substitutions needed. First, we broadly aligned entire transcriptions, then focused on individual utterances. By identifying utterance locations in the GS, we extracted corresponding segments from the ASR transcription. This 2-step approach enabled precise comparative analyses between the transcription sets.

### Evaluating the performance of ASR systems

To evaluate ASR system accuracy, we used 3 methods: WER,^16^ linguistic inquiry and word count^17^ (LIWC), and analysis of specific speech elements, including repetition, filler words, and proper nouns. Word error rate measures accuracy by counting transcription errors, including substitutions, deletions, and insertions. Linguistic inquiry and word count reflects the system’s ability to preserve spoken word nuances across language dimensions. Analyzing specific speech elements assesses the ASR’s capability to capture natural speech flow and details related to patient engagement, cognitive impairment, and mental disorders. These metrics are important for determining ASR performance, especially in accurately transcribing patient-nurse communication.

#### Calculating ASR errors using WER

Word error rate^16^ is a standard metric for assessing ASR system accuracy by comparing ASR-generated text with a reference transcription. It identifies errors classified into substitutions, deletions, and insertions.

**Figure 1.**
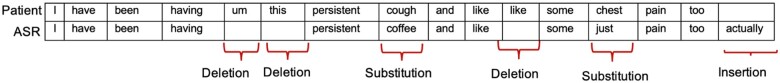
Visualization of WER components: substitutions, insertions, and deletions. Abbreviation: WER, word error rate.

The formula to calculate WER is as follows:


$$\mathrm{WER}=100\times \left.\left(\frac{S+D+I}{N}\right.\right)$$

where *S* represents the number of substitutions, *D* the number of deletions, *I* the number of insertions, and *N* the total number of words in the reference text. Figure 1 illustrates a WER calculation example where an ASR system incorrectly inserts “actually,” substitutes “cough” and “chest” with “coffee” and “just,” and deletes “this” and “um.”

We calculated WER by comparing patient utterances in GSs against ASR outputs. The average WER across all utterances was reported, with separate computations to assess differences between Black and White patients.

To evaluate the statistical significance of WER differences between ASR systems for Black and White patients, the Brunner-Munzel^18^ test was employed. This nonparametric test is particularly advantageous as it does not require the assumption of similar distribution shapes or equal variances among the groups being compared, offering a more suitable and robust analytical approach for datasets where traditional assumptions of normality and homogeneity of variances do not apply.

#### Calculating ASR errors using LIWC

The LIWC 2015 tool^17^ is a text analysis tool that classifies words across 10 multiple psychologically meaningful dimensions, including (1) Language structure, (2) Affect, (3) Social processes, (4) Cognition, (5) Perception, (6) Conversational dynamics, (7) Time orientation, (8) Physical states, (9) Drives, and (10) Lifestyle. Each dimension includes specific linguistic features—for example, terms indicating tentativeness or certainty are classified under cognition, whereas those related to sentence structure fall within language structure. Linguistic inquiry and word count has been validated in numerous studies for its ability to detect semantic and psycholinguistic cues associated with mental and neurological disorders.^4^^,^^5^^,^^19^

In this study, we used the LIWC tool to evaluate the ASR system’s accuracy in replicating human language complexity across 10 dimensions compared to manual transcriptions. Discrepancies in LIWC scores for the cognition dimension indicate the ASR’s difficulty in capturing cognitive nuances. Similarly, discrepancies in the Affect dimension highlighted challenges in capturing emotional cues like tone and word choice indicating positive or negative emotions. We calculated the percentage change in LIWC scores between ASR and GS transcriptions for each linguistic feature using the following formula:


$$\mathrm{Percentage}\;\mathrm{change}\;\mathrm{in}\;\mathrm{LIWC}\;\mathrm{score}\;=\;\frac{{\mathrm{LIWC}\;\mathrm{score}}_{\mathrm{ASR}\;}-\;{\mathrm{LIWC}\;\mathrm{score}}_{\mathrm{GS}\;}\;}{{\mathrm{LIWC}\;\mathrm{score}}_{\mathrm{GS}\;}}\;\times \;100$$

To determine LIWC scores at the dimension level, we first aggregated the scores for all linguistic features within each dimension. We then calculated the percentage change in scores between GS and ASR transcriptions for each dimension:


LIWC score for a dimension = ∑(Scores of all lingusitic features in the dimension)

$$\mathrm{Percentage}\;\mathrm{change}\;\mathrm{for}\;a\;\mathrm{dimension}\;=\frac{\;{\mathrm{LIWC}\;\mathrm{score}}_{\mathrm{ASR},\;\mathrm{dimension}}-{\mathrm{LIWC}\;\mathrm{score}}_{\mathrm{GS},\;\mathrm{dimension}\;}\;}{{\mathrm{LIWC}\;\mathrm{score}}_{\mathrm{GS}}}\times 100$$

#### Calculating ASR errors for specific speech elements: repetition, filler words, and proper nouns

We manually annotated 860 utterances for filler words, repetition, and medical and nonmedical proper nouns. To ensure consistency and validity, we developed a qualitative codebook with speech analysis experts, including detailed definitions and examples. An experienced annotator with a clinical nursing background annotated the entire dataset, while a second annotator independently reviewed a randomly selected 30% subset (about 258 utterances) to ensure reliability. Weekly discussions with the research team addressed inconsistencies and refined the coding process. The reliability between annotators was measured using the Kappa statistic, achieving a value of 0.89, indicating high agreement.

##### Filler words

Automatic speech recognition systems often misidentify filler words due to their variable tone, length, and volume. Additionally, ASR systems may erroneously insert filler words due to inconsistencies in speech patterns or misinterpretations of background noise as speech. We analyzed ASR transcriptions of filler words at the utterance level and normalized them according to a GS dictionary. For instance, variations of “um,” such as “umm” or “ummm,” were normalized to “um”. We then used the following formula to calculate the error rate:


$$\mathrm{Filler}\;\mathrm{word}\;\mathrm{error}\;\mathrm{rate}=\frac{\sum \left(\frac{{\mathrm{Incorrect}\;\mathrm{identified}\;\mathrm{filler}\;\mathrm{words}}_{\mathrm{ASR}\;}}{{\mathrm{Total}\;\mathrm{filler}\;\mathrm{words}\;\mathrm{per}\;\mathrm{utterance}}_{\mathrm{GS}}}\right)+{\mathrm{Utterances}\;\mathrm{with}\;\mathrm{inserted}\;\mathrm{filler}\;\mathrm{words}}_{\mathrm{ASR}}\;}{{\mathrm{Total}\;\mathrm{utterances}\;\mathrm{reviewed}}_{\mathrm{GS},\;\mathrm{ASR}}}\times 100$$

The numerator includes 2 components: first, the sum of the proportions of filler words incorrectly identified by the ASR system—whether by substituting them with incorrect words or omitting them entirely—relative to the actual number of filler words per utterance in the GS; second, the count of utterances in which the ASR system incorrectly added filler words, with each counted as a single error. The denominator is the total number of utterances reviewed, encompassing all utterances from both the GS and the ASR.

##### Repetitions

Automatic speech recognition systems frequently fail to accurately detect repeated words due to their reliance on statistical models predicting speech patterns. Rapid repetitions may be misclassified as anomalies or background noise, resulting in their omission from transcriptions. On the other hand, audio issues like echo or reverberation can inadvertently cause ASR systems to repeat words. Also word substitutions—such as transcribing “oh, no” as “no, no”—can introduce repetition. In this study, we quantified repetitions numerically; for example, “I know I know” was counted as 1 repetition, while “no, no, I forgot the appointment, no, no” was counted as 2.


$$\mathrm{Repetition}\;\mathrm{error}\;\mathrm{rate}=\frac{\sum \left(\frac{{\mathrm{Incorrect}\;\mathrm{identified}\;\mathrm{repetition}}_{\mathrm{ASR}\;}}{{\mathrm{Total}\;\mathrm{repetition}\;\mathrm{per}\;\mathrm{utterance}}_{\mathrm{GS}}}\right)+{\mathrm{Utterances}\;\mathrm{with}\;\mathrm{inserted}\;\mathrm{repetition}}_{\mathrm{ASR}}\;}{{\mathrm{Total}\;\mathrm{utterances}\;\mathrm{reviewed}}_{\mathrm{GS},\;\mathrm{ASR}}}\times 100$$

The numerator of the formula includes 2 components. The first component sums the proportion of repetitions that the ASR system incorrectly identified, relative to the actual number of repetitions per utterance in the GS. The second component counts the number of utterances in which the ASR system erroneously inserted repetitions, with each instance counted as a single error. The denominator is the total number of utterances reviewed, which includes all utterances from both the GS and the ASR.

##### Proper nouns

Automatic speech recognition systems often misinterpret proper nouns due to underrepresentation in training datasets and lack of contextual clues. For example, ASR systems may inaccurately recognize terms like “Duane Reade” (the name of a pharmacy) “lipid profile,” and region-specific names like “Astoria” or “Flushing.” To assess ASR accuracy, we calculated the error rate by evaluating transcription performance on both medical and nonmedical proper nouns using the following formulas:


$$\mathrm{Error}\;\mathrm{rate}\;\mathrm{for}\;\mathrm{medical}\;\mathrm{proper}\;\mathrm{nouns}\;=\left.\left(1-\frac{{\mathrm{Correctly}\;\mathrm{identified}\;\mathrm{medical}\;\mathrm{terms}}_{\mathrm{ASR}}\;}{{\mathrm{Total}\;\mathrm{medical}\;\mathrm{terms}}_{\mathrm{GS}}}\right.\right)\times \;100$$

$$\mathrm{Error}\;\mathrm{rate}\;\mathrm{for}\;\mathrm{nonmedical}\;\mathrm{proper}\;\mathrm{nouns}\;=\left.\left(1-\frac{{\mathrm{Correctly}\;\mathrm{identified}\;\mathrm{nonmedical}\;\mathrm{terms}\;}_{\mathrm{ASR}}}{{\mathrm{Total}\;\mathrm{nonmedical}\;\mathrm{terms}}_{\mathrm{GS}}}\right.\right)\times 100$$

The error rate for medical proper nouns was calculated as the percentage of incorrectly identified medical terms based on the total in the GS. Similarly, we computed the error rate for nonmedical proper nouns using the same approach.

## Results

A total of 35 Black and White patients were recruited to participate in audio-recorded patient-nurse verbal communication: 16 (45.7%) Black and 19 (54.3%) White. The gender distribution remained balanced, with 45% (*n* = 16) female and 55% (*n* = 19) male. The average age of participants was 67.8 years (SD = 14.4). All participants identified as non-Hispanic Black or non-Hispanic White. Most (90%) had Medicare coverage, and 70% lived alone.

Home healthcare nurses assessed participants for sufficient hearing and speech abilities, ensuring they could independently engage in communication without caregiver support. Nurses also conducted cognitive evaluations to verify participants’ ability to comprehend and sign the consent form. Each patient repeated the consent information to demonstrate understanding. Nurses confirmed that participants possessed adequate cognitive capacity and health literacy to follow medical instructions independently. Although we did not collect specific data on education levels, all participants spoke English as their primary language. We did not gather information on the number of languages spoken.

Regarding health conditions, for the sample used for data analysis, none of the patients experienced a decline in their mental, emotional, or behavioral status during the study. One participant had peripheral vascular disease, 1 had cerebrovascular disease, 2 had chronic obstructive pulmonary disease, 1 had renal disease, and 1 had a history of cancer.

Table 1 presents the characteristics of audio-recorded patient-nurse communication in the sample. The average duration of these conversations was 15 minutes, with a range from 11 to 21 minutes. The median number of words spoken by each patient was 1186. Specifically, Black patients had a median of 1271 words, while White patients had a median of 1101 words.

**Table 1.** Characteristics of audio-recorded patient-nurse communication.

	Average (SD)	25% Quartile	50% Quartile	75% Quartile
Length of audio-recorded Black verbal communication (measured in minutes)	15 (3)	12	16	17
Length of audio-recorded White verbal communication (measured in minutes)	16 (4)	15	16	18
Number of utterances by Black patients in each encounter in the manual transcription	95 (17)	95	97	101
Number of utterances by White patients in each encounter in the manual transcription	77 (24)	69	70	97
Number of words (tokens) spoken by Black patients in an encounter	1215 (425)	829	1271	1538
Number of words (tokens) spoken by White patients in an encounter	971 (569)	393	1101	1500

### ASR system performance: a comparative analysis using WER

**Figure 2.**
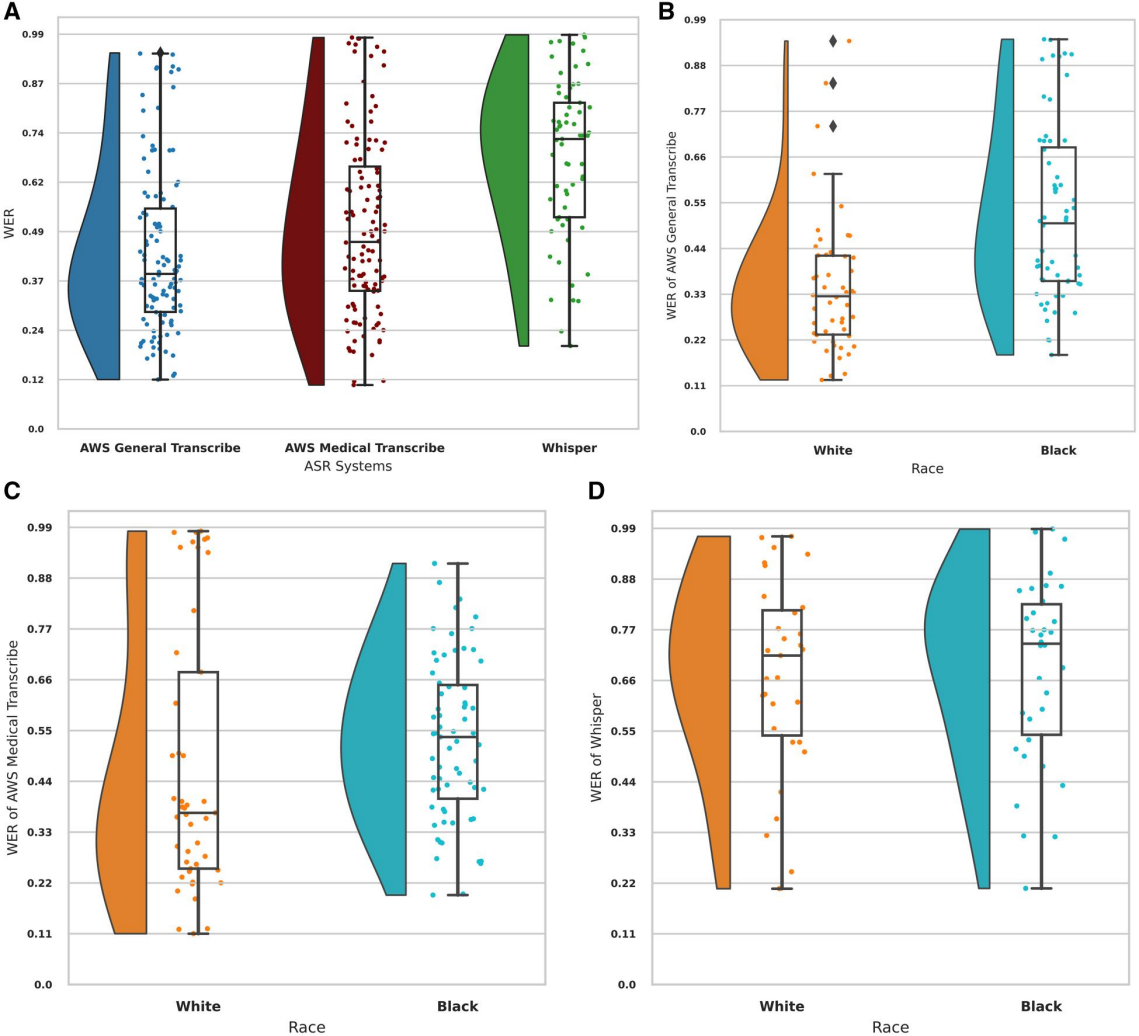
Comparative analysis of WER by race across different ASR systems. (A) WER distributions for AWS General Transcribe, AWS Medical Transcribe, and Whisper, highlighting differences by race. (B) WER for AWS General Transcribe, comparing performance between Black and White patients. (C) Similar breakdown for AWS Medical Transcribe. (D) Whisper, again comparing WER between Black and White patients. These visualizations underscore the differences by race in ASR transcription accuracy across various systems. Abbreviations: ASR, automatic speech recognition; AWS, Amazon Web Services; WER, word error rate.

Word error rate for ASR systems varied significantly: AWS General Transcribe had an average WER of 59% (median 39%, range [12%, 95%]), AWS Medical had an average of 62% (median 47%, range [11%, 99%]), Whisper averaged 84% (median 73%, range [21%, 99%]), and Wave2Vec had the highest average WER of 99% (median 91%). A lower WER indicates better performance and a higher WER indicates poorer performance. Due to Wave2Vec’s poor transcription quality and frequent out-of-dictionary words, it was excluded from further analysis. Figure 2A visualizes the distribution of WERs using density and box plots for AWS General, AWS Medical, and Whisper.

Further analysis across racial groups, using *Brunner-Munzel test*, showed all systems performed better in transcribing verbal communications from White patients compared to Black patients. Amazon Web Services General had the most pronounced difference, with a median WER of 33% for White patients vs 50% for Black patients, as shown in Figure 2B (*P* = 0.016). Amazon Web Services Medical had a median WER of 37% for White patients and 54% for Black patients, as shown in Figure 2C (*P* = 0.0012). Whisper showed the smallest difference, with median WERs of 72% for White patients and 75% for Black patients, as shown in Figure 2D (*P* = 0.66).

Additionally, significant challenges for ASRs transcriptions emerged with short utterances. Specifically, for AWS General Transcribe, the top-performing ASR system, the average (of) WER was high at 86% for utterances under 5 words, decreased to 51% for utterances of 5 to 11 words, and further reduced to 37% for those over 11 words. These findings underscore the difficulties of ASR systems encounter in accurately transcribing brief verbal exchanges.

### ASR system performance by LIWC category

**Figure 3.**
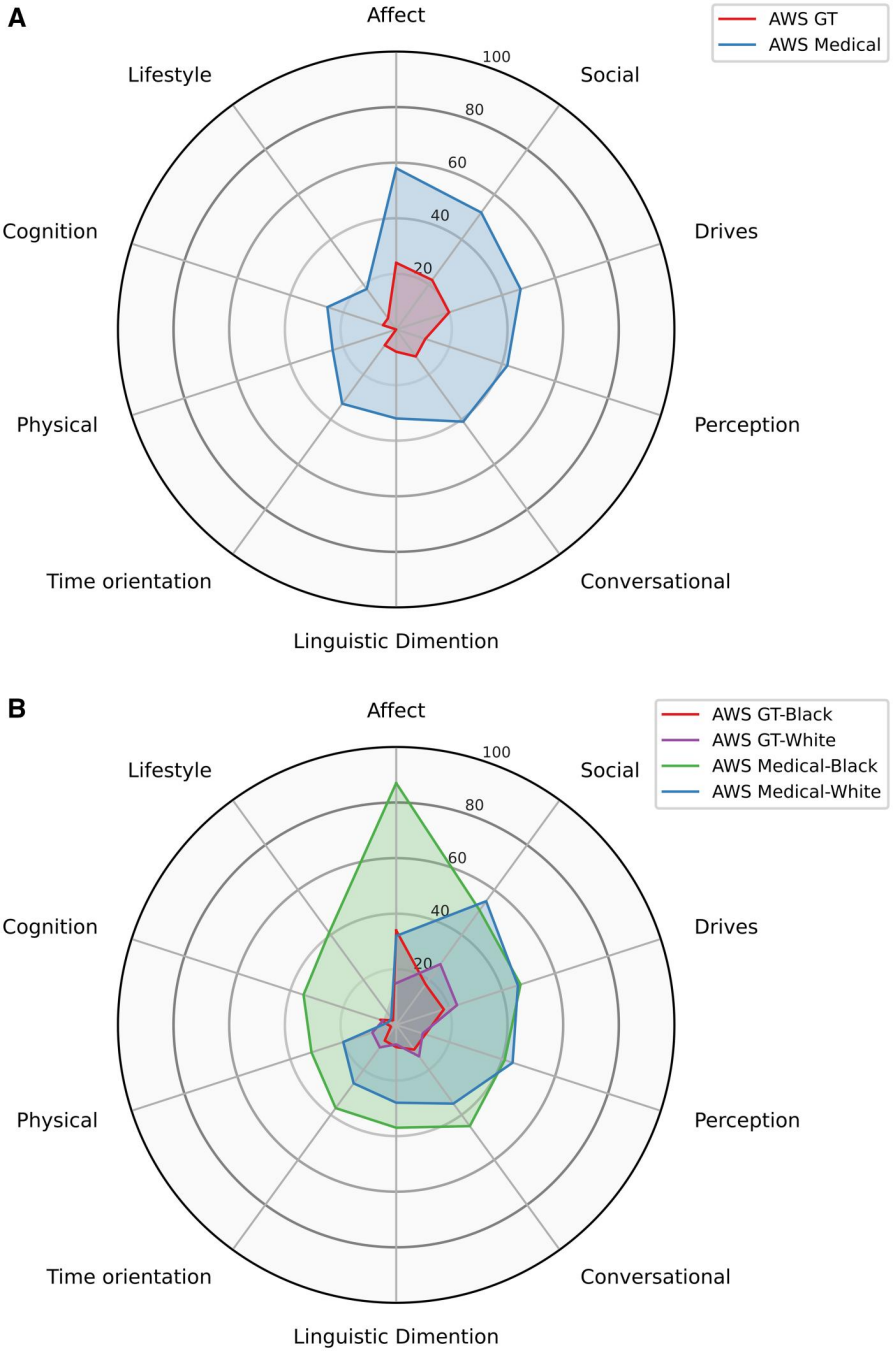
Radar plots showing the percentage changes in LIWC scores of AWS General Transcribe and AWS Medical in various LIWC linguistic dimensions. (A) Overall percentage changes across all dimensions for the sample of the study. (B) Percentage changes for Black and White patients. Abbreviations: AWS, Amazon Web Services; LIWC, linguistic inquiry and word c ount.

Figures 3A shows the radar plots comparing AWS General and Medical transcribe performance across 10 linguistic dimensions defined by LIWC. Amazon Web Services Medical, with a higher WER, shows substantial inaccuracies, especially in “Affect,” “Social,” “Drivers,” “Perception,” and “Conversational” dimensions. Amazon Web Services General, with the lowest WER, exhibits significantly smaller deviations across 10 dimensions, with small discrepancies in “Physical,” “Time Orientation,” “Cognition,” and linguistic dimensions.

Figure 3B highlights the differences by race: AWS Medical displays greater inaccuracies in the dimensions of “Affect,” “Lifestyle,” and “Cognition” for Black patients compared to White individuals. Although AWS General demonstrates fewer differences by race, it follows a similar trend, with notable inaccuracies primarily in the “Affect” dimension. However, discrepancies in the “Social” and “Drive” dimensions are slightly more pronounced for White patients. Both AWS General and Medical consistently show the largest discrepancies in the “Affect” dimension for Black patients.

Whisper was excluded from the LIWC scores analysis due to its higher WER and substantial deviation in LIWC scores from the GS, and thus, we focused on the comparative performance of the 2 leading ASR systems.

**Figure 4.**
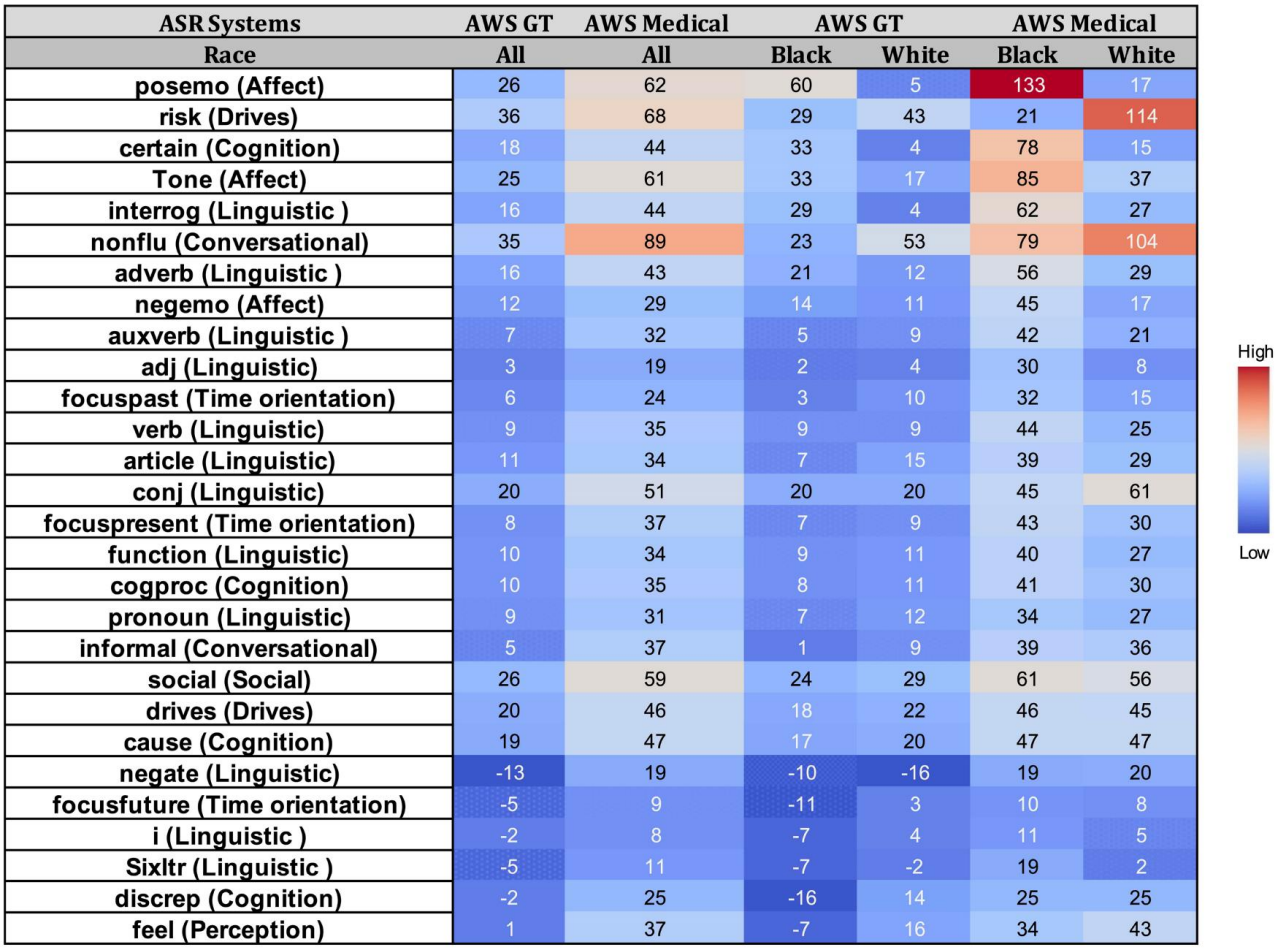
A heatmap visualizing percentage changes in LIWC scores of AWS General and AWS Medical transcriptions from gold standard LIWC scores across selected linguistic features, with breakdowns for Black and White patients. Warmer colors indicate larger discrepancies (lower accuracy), while cooler colors indicate smaller discrepancies (higher accuracy).

Figure 4 presents a heatmap visualizing percentage changes in LIWC scores of AWS General and AWS Medical transcriptions from GS LIWC scores across selected linguistic features, with breakdowns for Black and White patients. Amazon Web Services General exhibited pronounced overestimations (positive percentage changes), particularly in the Affect dimension with overestimations in “Positive Emotion,” “Tone,” and “Negative Emotion,” and in the Drives dimension for “Risk.” These overestimations were more prominent in Black patients due to insertion or substitution errors. In contrast, the nonfluencies feature (linguistic dimension) showed overestimations affecting White patients. Amazon Web Services General also showed negative percentage changes (underestimations) in “Negate” (Linguistic), “Focus Future” (Time Orientation), and “words longer than 6 letters” (Linguistic), predominantly affecting Black patients. Amazon Web Services Medical, on the other hand, tends to overestimate these categories, indicating insertion or substitution errors.

### Measuring accuracy of ASR systems for specific speech elements: filler words, repetition, and proper nouns

**Figure 5.**
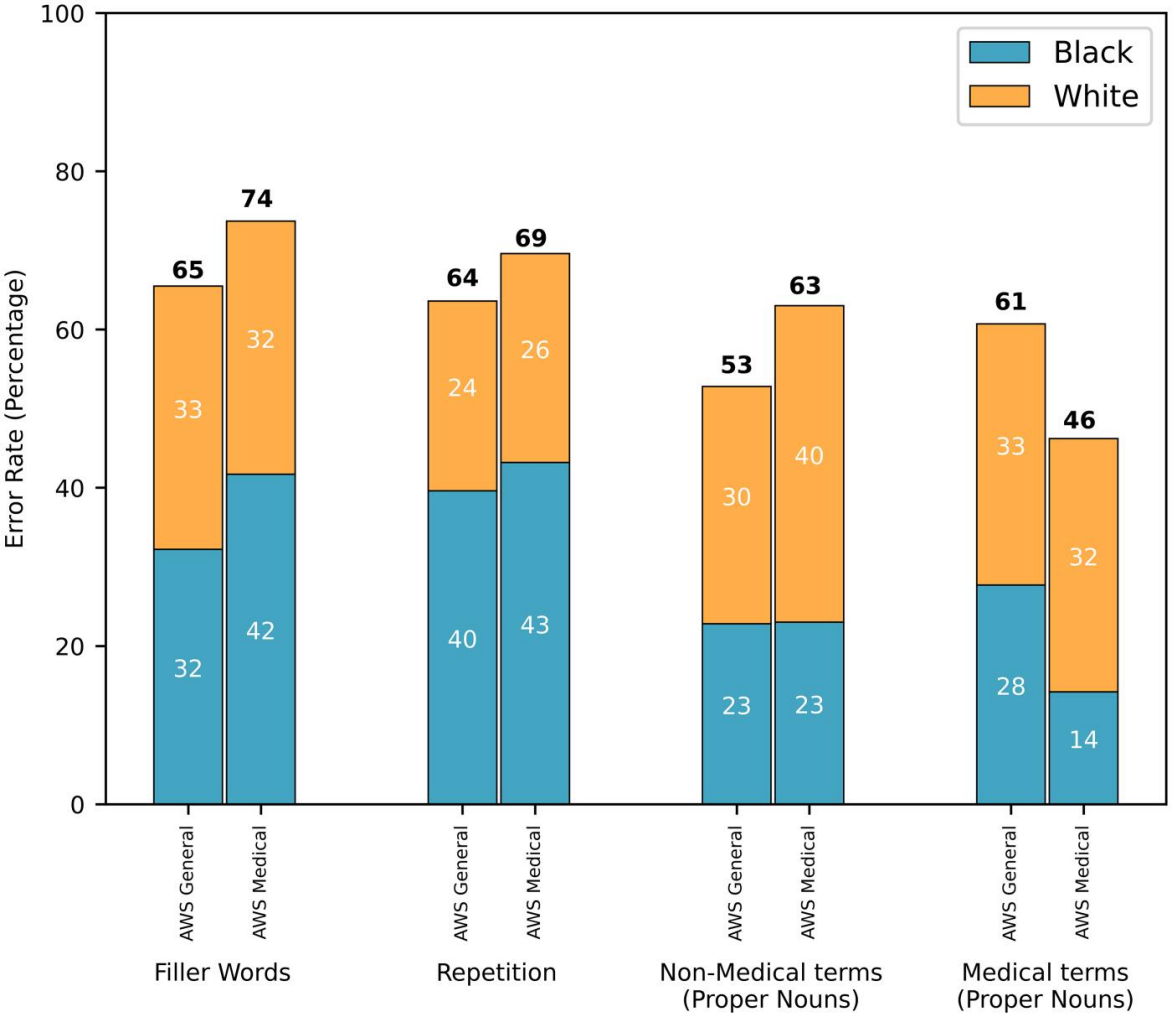
Comparing the error rate (%) of both AWS General vs AWS Medical, in detecting different speech elements (filler words, repetitions, nonmedical and medical proper nouns) when compared to the gold standard (manual transcription). Abbreviation: AWS, Amazon Web Services.

Among the 860 GS utterances, 146 (17%) contained filler words like “um,” “uh,” and “oh.” “Um” was mainly used by Black patients, while White patients used “oh” and “uh” more frequently. Amazon Web Services General correctly recognized 62 of these utterances but misidentified 84 (54 correctly recognized, 89 misidentified for AWS Medical). Additionally, AWS General mistakenly recognized 77 utterances as containing filler words when there were none (166 for AWS Medical). Figure 5 shows the error rates for filler words, which were higher for Black patients compared to White patients.

Out of 121 utterances with repetition, AWS General correctly identified 37 (44 for AWS Medical), misidentified 77 utterances (84 for AWS Medical), and mistakenly recognized 49 utterances with repetition when none existed (54 for AWS Medical). This resulted in error rates of 65% for AWS General and 74% for AWS Medical, with higher rates in Black patients compared to White patients for AWS Medical. However, AWS General showed almost the same error rate for both groups (Figure 5).

In the GS, there were 21 medical proper nouns, such as “Tylenol” and “Xanax.” The error rate was 61% (13/21) for AWS General and 46% (10/21) for AWS Medical. For 52 nonmedical proper nouns, like “Sandy” and “Pennsylvania,” the error rate was 53% (28/52) for AWS General and 63% (33/52) for AWS Medical. Compared to filler words and repetition, the error rates for medical and nonmedical terms were higher for White patients compared to Black patients (Figure 5). More information is presented in Appendix S2.

## Discussion

This study is the first to systematically assess the accuracy of AWS General, AWS Medical, Whisper, and Wave2Vec in transcribing patient-nurse communications in HHC, focusing on differences between Black and White patients. Transcriptions were compared against a rigorously developed manual GS. Amazon Web Services General achieved the highest overall accuracy with an average WER of 59% (median 39%), having higher performance in specific speech elements, including repetition (error rate = 64%), filler words (error rate = 65%), and nonmedical proper nouns (error rate = 53%). Amazon Web Services Medical outperformed in medical proper nouns with an error rate of 46% vs 61% for AWS General. All ASR systems struggled with short utterances. Specifically, AWS General had a WER of 86% for utterances under 5 words, compared to 37% for those over 11 words, mainly due to diarization issues where even correctly captured words were assigned to the wrong speaker, often in fast-paced or overlapping dialogue.

Whisper and Wave2Vec showed lower transcription accuracy in verbal communication, with median WERs of 84% and 91%, respectively. This gap stems from differences in training datasets and model architectures. Whisper’s transformer-based model struggles with colloquial language and specialized medical terms due to limited domain-specific data. Wave2Vec, using self-supervised learning on large amounts of unlabeled audio, has less accurate language modeling compared to systems trained on extensive labeled datasets. Unlike Wave2Vec, which is fine-tuned on smaller datasets like LibriSpeech, AWS services use vast labeled datasets that include diverse linguistic patterns and specialized terminology. This comprehensive training enhances the transcription accuracy of AWS General and Medical in HHC contexts. However, AWS Medical’s lower accuracy compared to AWS General is due to its training on healthcare professional language, whereas AWS General’s training on colloquial language aligns better with patient communication in HHC.

All ASR systems evaluated exhibited lower transcription accuracy for Black patients relative to White patients, with significant discrepancies particularly notable in AWS General and Medical Transcribe. Given that there is a difference in accuracy that likely stems from limited diversity in training datasets and inherent biases during the model development process in what are now widely available and implemented systems, it is suggested that this study has identified an under-recognized racial disparity with substantial relevance to disease detection and healthcare delivery. Previous studies also support these findings: Koenecke et al^20^ observed that error rates for African American speakers were nearly double those of White speakers in commercial ASR systems. Further studies by Tatman^21^ and Wassink et al^22^ highlighted decreased performance on dialects and accents underrepresented in training data, specifically African American, Chicano, and Native American. Furthermore, the diary study by Mengesha et al^23^ revealed the profound psychological impact of ASR inaccuracies on African American users, who experienced feelings akin to discrimination due to frequent ASR errors.

Building on this, Kulkarni et al^24^ further demonstrated that demographic disparities extend beyond race to factors such as gender, age, skin tone, and geographic region. Their study on ASR systems (Whisper and MMS [Massively Multilingual Speech, a speech recognition system developed by Meta]) in Portuguese-language conversations revealed significant disparities in transcription accuracy, which were partially mitigated using techniques like oversampling. Similarly, Graham and Roll^25^ focused on English accents and showed that Whisper performs better with native English accents like American and Canadian, while nonnative accents, particularly tonal languages like Vietnamese, face higher WERs. Both studies emphasize how demographic traits—including language proficiency and geographic location—significantly affect ASR accuracy, reinforcing that biases are not confined to 1 domain or language group. These findings collectively underscore the need for broader and more diverse training datasets to reduce systemic ASR disparities, particularly in high-stake environments like healthcare, where these disparities can lead to misdiagnoses and compromised patient outcomes.

In our analysis using the LIWC tool to evaluate linguistic dimensions, we observed significant disparities in ASR system performance, particularly with AWS General and Medical Transcribe. These systems showed notable inaccuracies when transcribing emotional terms, tone, expressions of risk, and cognitive markers such as uncertainty. Transcription accuracy for Black patients was consistently lower, highlighting racial disparities in ASR system performance. Errors were especially frequent in key speech elements like filler words and repetition, which are important indicators of speech fluency and reflect cognitive processes and emotional states. These transcription inaccuracies can distort the emotional context and intent of communication, potentially leading to misdiagnoses or misinterpretations of the patient’s condition, which may result in inappropriate treatment decisions or overlooked symptoms.

Despite these overall disparities, the ASR systems performed better in detecting specific speech elements—such as filler words and repetitions—in Black patients’ speech. This suggests that while general transcription accuracy is lower, certain linguistic features are transcribed with more accuracy. These findings underscore the need for further refinement of ASR systems to enhance their ability to handle diverse speech patterns, ensuring more equitable performance across all racial groups.

Audio recording of patient-nurse communication is not currently standardized in clinical workflows. To address this, we conducted pilot studies to identify effective audio-recording procedures. The findings showed that both patients and nurses were comfortable with the process, and patients found it a potentially useful tool for reviewing clinicians’ instructions. Involving healthcare stakeholders, especially HHC clinicians and managers, is an important process for integrating audio recording into workflows and establishing necessary processing methods.^6^ With advancements in AI technology, HHC workflows could soon include risk prediction models based on these recordings, enhancing patient risk identification. This study highlights the critical need to address transcription accuracy disparities for Black patients before fully integrating these technologies into healthcare settings.

Systematic disparities in ASR systems can have profound and far-reaching negative implications.^26–28^ These disparities often disproportionately affect minority populations, as ASR systems may perform poorly with diverse dialects.^29^ Inaccurate transcriptions of minority dialects caused by ASR disparities can jeopardize patient safety by leading to miscommunication, misdiagnoses, and inappropriate treatment plans. For example, misrecognizing medication names or dosages may result in serious medication errors. Erroneous documentation creates administrative burdens and increases operational costs by requiring corrections. This can result in clinician frustration and burnout, ultimately impeding the adoption of beneficial technologies.^30^ Furthermore, ASR errors that misrepresent patient dialects can damage the foundational trust in patient-provider relationships, hindering effective communication and patient engagement.^31^^,^^32^ Ethical concerns arise regarding fairness and justice, emphasizing the imperative to prevent technological advancements from inadvertently harming vulnerable populations or contributing to systemic inequities.^32^

This study is not without limitations and is constrained by several factors. First, it includes a small sample size of utterances from patient-nurse interactions, which were selected to be typical of such communications; however, offer limited sample size. However, the alignment of the results with those reported from previous studies suggests that the transcription accuracy issues identified are part of a broader trend affecting ASR systems in healthcare. Second, the data, sourced from a single New York City HHC provider, may not reflect the regional linguistic diversity found across the United States. Nonetheless, New York City is an ideal place to conduct this line of work given that it is a “melting pot” of myriad speech patterns and vernaculars reflecting years of human migration patterns, variability of ages in which they first started living in New York City (and acquiring language), and variability among residents living in communities for a wide range of years over which time language patterns may become more fixed. Further, although 4 commonly used ASR systems were analyzed, other popular ASR systems like Google and Microsoft were excluded. Finally, metrics such as phoneme error rates, semantic error rates, and speaker diarization accuracy, which could enrich understanding of ASR performance, were not assessed but are of interest in exploring these points in larger datasets.

## Conclusion

This study highlights significant disparities in the accuracy of ASR systems, particularly among Black and White patients, underscoring the critical need for improvements in these technologies. It is imperative that ASR systems do not exacerbate existing healthcare disparities. Therefore, they must be rigorously tested and refined to handle a diverse array of speech patterns and accurately transcribe across different demographic contexts. Only through such dedicated enhancements can ASR systems be responsibly integrated into healthcare settings, ensuring they support accurate clinical assessments and equitable patient care.

## Supplementary Material

ooae130_Supplementary_Data

## Data Availability

The data was collected from VNS Health, a homecare agency located in New York. According to IRB protocol, we are not able to share the data publicly. The code is available in the following link: https://github.com/NeuroTechAnalytics/speech-bias-study.
