# Supplementary material for: Decoding disparities: evaluating automatic speech recognition system performance in transcribing Black and White patient verbal communication with nurses in home healthcare
Source: JAMIA Open. 2024 Dec 10;7(4):ooae130. doi: 10.1093/jamiaopen/ooae130 (PMC11631515; doi:10.1093/jamiaopen/ooae130)
Supplement: ooae130_Supplementary_Data [file ooae130_supplementary_data.docx]

**Appendix A:**  Description of the characteristics of each Automatic Speech Recognition System. (AWS GT: Amazon General Transcribe; AWS Medical: Amazon Medical Transcribe; ASR: Automatic Speech Recognition)

| ASR | Description |
| --- | --- |
| AWS GT ^35^ | Amazon Transcribe's architecture includes a comprehensive speech recognition pipeline. Users provide audio input in formats such as MP3, WAV, and FLAC, which undergoes preprocessing for noise reduction, normalization, and segmentation. Acoustic features like mel-frequency cepstral coefficients (MFCCs) and linguistic features capturing phonemes and words are extracted. Deep learning models, including the acoustic model (mapping features to phonetic units), the language model (providing context for improved accuracy), and the speaker diarization model (distinguishing different speakers), are utilized. The decoding process combines these model outputs to convert audio to text, with post-processing enhancing readability through punctuation and error correction. Additionally, Amazon Transcribe supports a customizable dictionary for specific jargon and terminology, trained on a diverse dataset that includes various accents and languages to ensure accurate transcription. |
| AWS Medical ^36^ | AWS Medical Transcribe follows a similar architecture to Amazon Transcribe, however, it is specifically trained on medical text and terminology from various fields such as Cardiology, Radiology, and Oncology. This speciality in training is expected to enhance its accuracy in transcribing specialized medical terminology. It also offers the same features of AWS GT, such as speaker diarization. Leveraging AWS's robust cloud infrastructure, AWS Medical Transcribe ensures high scalability and strong security measures, including data encryption and compliance with healthcare industry standards. This specialized training is anticipated to make it highly suitable for medical applications, providing reliable and accurate transcription services tailored to the needs of medical professionals. |
| Whisper ^37^ | Whisper's ASR architecture, developed by OpenAI, adeptly manages a broad range of audio formats. It preprocesses audio by performing noise reduction, normalization, and segmentation into smaller, manageable segments. Utilizing a unified deep learning approach, Whisper incorporates a large transformer model that integrates both acoustic and linguistic information directly from the waveform, eliminating the traditional separation of acoustic and language models. Additionally, Whisper is trained on a diverse, multilingual dataset that includes 100 languages, enabling effective handling of various accents, dialects, and languages. The model is robust against various types of background noise and different audio qualities, making it ideal for real-world applications. Although it primarily focuses on transcription, Whisper's architecture includes some capabilities for speaker differentiation, though not as extensive as dedicated speaker diarization models. Users can also fine-tune Whisper on domain-specific data to improve accuracy for specialized applications or jargon, akin to customizable dictionaries in other ASR systems. conditions. |
| WAV2Vec 2.0 ^38^ | Wav2Vec 2.0, developed by Meta AI, is an open-source ASR system that combines a small amount of labeled data with a large corpus of unlabeled audio for training, reducing dependency on labeled data by learning directly from waveform patterns. Users provide raw audio input, which is minimally preprocessed. The system employs a convolutional neural network (CNN) to encode raw audio into latent representations. These representations are processed by a transformer model, capturing contextual information through self-attention mechanisms. Pre-trained on extensive unlabeled audio data, the model has been fine-tuned with a smaller labeled dataset like LibriSpeech. The transformer’s output is decoded into text, and post-processing enhances readability with punctuation and error correction. Wav2Vec 2.0 generalizes well across languages, even with limited annotated resources, leveraging extensive unlabeled speech data for improved performance and versatility. |

**Appendix B. Comparison of Utterance Instances in ASR systems and Gold Standard Transcriptions for Filler and Repetition Words**

|  | **ASR and Gold Standard had zero instances** | **ASR and Gold Standard had the same instances** | **ASR had less instances than Gold Standard** | **ASR had more instances than Gold Standard** |
| --- | --- | --- | --- | --- |
| **Comparing instances of utterances with filler words in gold standard and ASR transcriptions** | | | | |
| **AWS GT** | 641 | 62 | 62 | 99 |
| **AWS Medical** | 571 | 54 | 57 | 182 |
| **Comparing instances of utterances with repetition words in gold standard and ASR transcriptions** | | | | |
| **AWS GT** | 694 | 44 | 64 | 62 |
| **AWS Medical** | 691 | 37 | 73 | 65 |
